# Academy of Stomatology

**Published:** 1897-01

**Authors:** 

**Affiliations:** Academy of Stomatology


### ACADEMY OF STOMATOLOGY.

A REGULAR meeting of the Academy of Stomatology was held in the rooms of the society, November 24, 1896, Dr. James Truman presiding. The routine business being despatched, the report of the Clinic Committee was called for. Dr. H. C. Register, chairman of the committee, stated that the subjects of the clinic of the afternoon were the use of Fowler's solution as an obtundent, and the cleansing of the teeth with the aid of the tincture and compound tincture of iodine (liquor iodi compositus, Lugol's solution). This subject was to have been expounded and the method demonstrated by Dr. Francis, of New York, who forwarded a letter of regret at his inability to attend.

He states that he invariably uses iodine; it softens mucous accumulations, and acts as a tell-tale for disguised deposits of calculi. The preparation employed is the compound tincture of iodine. The method has been frequently described by Dr. Francis, and so he omits a detailed description of it in his letter.

Dr. Register described the method of using iodine, which is as follows: Applications of the dilute tincture are freely made to teeth and gums, which not only constringe puffy gums, drawing them away from about the teeth, but clearly outline any deposits which may be present. Deposits are defined by this means, which without it would entirely escape detection. The patient expresses gratification, not annoyance, at the sensation produced by the drug. The iodine appears to have the power of loosening the deposits upon the teeth. It insinuates itself into the minute recesses of rough areas of the crowns. The iodine is followed by applications of commercial ammonia, which is immediately decomposed, a colorless solution formed, and the teeth are found to be much lighter in color. The surfaces are subsequently cleansed and polished by means of buffs and fine pumice.

*Dr. Jack.*—What is your method of applying Fowler's solution as an obtunding agent?

*Dr. Register.*—The mouth is first sterilized by washing with a ten-per-cent. solution of electrozone, and the rubber dam applied. The cavity is dried with bibulous paper, and an application of carbolic acid is made, which is followed by blasts of warm air; then by a hot alcohol blast; a chamber containing cotton saturated with alcohol is heated, and a current of air charged with the heated vapor is directed into the cavity. A pledget of cotton is dipped in the Fowler's solution and applied to the cavity walls, where it remains for about three to five minutes, when it is wiped away, and the hot blast applied for a minute or two.

*Dr. Darby.*—It appears to me that the measures you describe would be quite sufficient without the use of Fowler's solution. I should expect, after the adoption of such measures, that I could excavate almost any cavity without even discomfort to the patient.

*Dr. Huey.*—I would like to inquire how it is possible, where a great number of agents are combined or successively applied, to distinguish which of them has acted as the analgesic. I mean this not only in connection with the method described by Dr. Register, but with those preparations which contain a number of powerful coagulants and obtundents.

*Dr. Register.*—Several of the members have inquired into the use of the ribbon matrix, which I have been using for some time, and have demonstrated frequently. They are made of strips of thin, planished copper, thin enough to pass into small interspaces between the teeth, and be laced in and out these spaces. Cavities which are compound have by this means an additional wall made, against which the filling is impacted and its contour outlined. A great deal of time is saved by this means, not only in the impacting of the filling, but in the subsequent polishing operations.

*Dr. Guilford.*—I believe Dr. Herbst is the father of this form of matrix. He demonstrated its use several years ago when in this country. He used thin strips of steel or of German silver. One end of the strip was rolled into a cylinder, which anchored it between the teeth. The free end was then drawn into position and an additional wall given to the cavity.

*Dr. Register.*—I do not doubt this, but I did not see or hear of Dr. Herbst's matrix. Dr. Louis Jack is the father of the modern matrix, although Dr. William Trueman can furnish data which will show the matrix itself to be over one hundred years old.

*Dr. Burchard.*—Dr. Jack's matrix has a function distinct from all others, it outlines the desired contour; with previous matrixes and most others the contour is indefinite; with the Jack matrix the contour is predetermined and fixed, so that judicious burnishing of the filling proper condenses and shapes its cervical portion.

*President Truman.*—The paper of the evening, "Hypersensitivity of Dentine and its Treatment," by Dr. Burchard, is now in order.

*Dr. Burchard.*—I think I should preface the reading of this essay by an apology to the members for its elementary character, and the evidences in it of hasty preparation. I am so busy with other matters that I have had only time to bring the subject matter of the essay together and have not looked at it since, to correct or modify.

The treatment of the subject, designedly deals alone with the general principles involved; details and minutiæ are deliberately omitted, in the hope that other essays upon the same subject may treat each phase of it exhaustively. It was nothing but a sense of duty which induced me to provide an essay for this meeting, as I can ill spare the time from other literary work, but as we were short an essay for November, and the council has not had time to secure one, I have assumed that duty as a part of my duties as a member of the council.

(For Dr. Burchard's paper, see page 1.)

DISCUSSION.

*The President.*—In my opinion, no essay ever called for less apology than this of Dr. Burchard, it is clear, practical, and exhaustive. The subject is now open for discussion, I shall call upon Dr. Cryer.

*Dr. Cryer.*—Personally I am highly pleased with the essay. It covers all of the principles involved in the subject, so that I shall confine my discussion to but one phase of the matters treated,—that is, the ill effects of cocaine injections about the jaws. The essayist alludes to the dangers involved in this operation, but my experience indicates that he might emphasize them still more, for I have noted serious surgical troubles, which if not caused by the injections, certainly followed close upon them. I shall select a few examples for illustration. The first case was that of an adult male, who received a cocaine injection prior to the extraction of a lower third molar.

Twenty-four hours after the operation a sensory paralysis began, which quickly involved all of the branches of the third or inferior maxillary division of the fifth nerve of the side. Within a few days more the paralysis was found to involve the second division of the nerve, and shortly after the first division. Following upon this, evidences of involvement of the seventh nerve became marked, motor paralysis of the muscles of the face being noted. It was at this time that I was called to see the case and elicited the above history, and noted that a peculiar necrotic condition of the inferior maxilla existed; there was no evidence of sequestra being formed, the bone appearing to have undergone a molecular death which did not resemble ordinary caries, nor was there any evidence of osteomyelitis. The dead bone was burred away, and the case recovered, but the nerve lesion extended until the cerebrum was involved.

In another case, where a cocaine injection had been made over a molar, evidences of inflammatory disturbance in the second division of the fifth nerve became marked, and three months after, a necrosis similar to that described before was found.

I have seen nine other cases in which there were similar disturbances noted. To sum up, my experience is strongly against the use of cocaine injections, and when I wrote the chapter on extraction for the new text-book on operative dentistry, I refused to consider the subject of the hypodermic use of cocaine about the jaws except to condemn it; so that Dr. Burchard was assigned the work, and it is my opinion that he is also opposed to the practice.

*Dr. Burchard.*—These cases of Dr. Cryer indicate an ascending neuritis, and possibly are an evidence of the trophic influence of the nervous supply to a part. Were there any inflammatory disturbances preceding or attendant upon the progress of the necrosis, such as periostitis?

*Dr. Cryer.*—None that I noted.

*Dr. S. Freeman*, of New York.—The essayist has alluded to the several methods in which electricity is used as an obtundent. In a conversation with Dr. Flagg he stated that he had had enough of electricity. The apparatus of Flagg produces an interrupted current which differs in its effects from galvanism, as noted in the essay. I have used the cataphoric current and in a manner which lessens the time of inducing analgesia. The tooth is placed under dam, is dried, and a saline solution of cocaine is applied, which remains a short time; then the cataphoric current is applied, and is effective in about eight minutes.

*Dr. Inglis.*—The dental helix does not produce an induced current and is not analgesic. The positive electrode is placed on the gum over the tooth, the negative electrode is held in the hand; instead of producing analgesia it produces a diverting sensation which lessens the patient's perception of the pain of excavation.

The essayist has not alluded to the use of eucaine, which in two-per-cent. solution is more effective than cocaine, and produces local hyperæmia instead of local anæmia.

*Dr. William H. Trueman.*—Before discussing the essay I should prefer to read and reread it when it is printed, as I did not catch all of its bearings. As a matter of fact, I seldom use an obtundent, except properly-formed tools, and applying them with a view to the work they are to do and how they can do it. This is an important matter, and one to which too little attention is directed. Those of you who are familiar with lathe work know how much the cutting of a tool depends upon the precise shape of the edge of the tool, the way it is clamped in the slide-nest, and the way it is presented to the metal it is to cut. Correctly applied in all three of these particulars, a tool cuts smoothly; improperly formed or applied, it chatters, and instead of cutting smoothly it jars and cuts roughly. Again, the tool edge should be shaped with a recognition of the material it is to cut, and a tool made to cut or dress enamel has an entirely different edge and cutting angle from one designed to cut dentine. In cutting this tissue the tool edge should be shaped so that it will cut like the chisel of a carpenter's plane; shave off a smooth cut of dentine, not chatter or jar out pieces of it. A tool which cuts best is rigidly held to its work by an inflexible stem, and will produce the least pain, in most cases producing little or none.

I have used oil of mustard as an obtundent, and have found it to do its work well. It is very pungent and comparatively inexpensive. I do not know whether the essayist mentioned this agent.

*Dr. Burchard.*—No; nor did I mention the fact that it is a powerful germicide.

*Dr. Register.*—I have been using Fowler's solution as an obtundent for more than four years, and have noted no ill effects in connection with its use. I recognize, of course, that it would not do to teach students to use any of the arsenical preparations as obtundents, but as I am talking to experienced practitioners, I present the subject for your consideration. I do not believe a pulp can be killed by its use. I have tried it and failed; moreover, I have never had a case of pulpitis following its use. Of course, an antidote should be applied after using it. I have used Fowler's solution in double strength (two per cent.) and filled the cavity with cement, and after weeks, upon removing the filling have found the dentine sensitive. Our essayist before he became a professor was much taken up with this matter when I discussed it with him.

*Dr. Burchard.*—Cautiously curious is the phrase, not "taken up with it."

*Dr. Register.*—Yes, you were suspicious.

*Dr. Jack.*—It appears to me, Dr. Register, that your method consumes quite as much time as that required for inducing cocaine anæsthesia by means of electrical osmosis.

*Dr. Register.*—Yes, I think it does. I would like to test the comparative effectiveness of the two methods in point of time. It is to be understood, of course, that I advise extreme caution in the use of Fowlér's solution.

*Dr. Guilford.*—The paper is very exhaustive, covering all of the principles involved in the subject. My observations in this connection extend over a long period of years,—about thirty. Beginning then with keen edge instruments as obtundents, we come at this day to electrical osmosis, and what have we not had between? The demands for dentinal analgesia are, I believe, more imperative to-day than ever before. In my opinion, the most important element in inducing or producing this analgesia is desiccation. It is very rare that you will find a tooth which has had its dentine dried until it shows almost chalky white that will exhibit any sensitiveness. And, again, drying in connection with other obtundents renders these latter more effective. The milder obtundents are, as a rule, not positive enough in their effects. I have used sulphuric acid as an obtundent, and am glad the essayist has called pronounced attention to this agent, for it is of great importance. I use it in combination with glycerin, making a body corresponding with the proprietary agent known as Thayer's No. 4.

*Dr. Freeman.*—That preparation contains trichloracetic acid.

*Dr. Guilford.*—When the editor of the *Dental Cosmos*, Dr. Kirk, made analyses of a great number of proprietary agents, he stated that sulphuric acid was the acid in this preparation. I find this combination of glycerin and sulphuric acid to be excellent and prompt in its effects. Certainly cataphoresis is the best of all our means, as it does the work without producing chemical destruction, and does it thoroughly; the time required is the only objection. I think Dr. Van Woert, of Brooklyn, first called attention to sulphuric acid as an obtundent. The effect of sulphuric acid is self-limited.

*Dr. Jack.*—What is the technique?

*Dr. Guilford.*—The rubber dam is applied, the cavity dried, and the softer caries removed; then dry again, and dip a pin-head piece of cotton in the acid and lay it upon the dentine, and touch the dentinal walls, in five minutes the excavation may be made. As a rule, one application is sufficient.

*Dr. Freeman.*—Some years ago Dr. Herbst used a saturated solution of cocaine in sulphuric acid as an obtundent. I have heard operators who have used sulphuric acid as an obtundent complain of the caving in of fillings placed in the cavities, and again, in using sulphuric acid in the roots of teeth, we may erode the roots and make large openings through them at the apex. I have used Thayer's preparation in root-canals, this preparation containing trichloracetic, not sulphuric, acid.

*A. H. Porter.*—The essayist, ably representing current thought, expressly implied its inability to express an accurate explanation of exaltation and nutrition. Electro-physiology and electro-chemistry, measuring vital action with mathematical precision, attack every inch of the ground, and determine at what point a stimulus increases instead of diminishes pain.

The specific elements of a cell combine to give it a specific tension, enabling it to respond to specific pressure (vibrations of air, chemical agents, ethereal oscillations, etc.). In physical language this response is effected by the release of the tension, chemically it is a dissociation. But this is not the only effect of the primary stimulus from the environment. Dependent on the dissociation of the exalted cell, an inverse association from blood plasma or adjacent tissue takes place. If the primary stimulus ceases at the moment of complete dissociation (a critical point to the organism), and the new cells are in excess of the disintegration, the effect has been constructive or anabolic. At this juncture the dissociated energies, recognized as carbonic acid gas, urates, etc., expel themselves.

When, however, by the continuance of the primary stimulus they cannot accomplish this, their force is exerted against the surrounding cells, causing the intense pain, overwhelming sense of pressure, failure of circulatory and respiratory activities characteristic of the prolonged use of anæsthetics and poisons. The hallucinations described by subjects are due to the dissociation of a cell by another instead of by an excitation from the outer world.

This antagonistic pressure is well illustrated in the many and varied forms of disease,—cancers, tumors, microbes and their products, concretions, gaseous accumulations, and morbid growths more or less organized.

*Dr. Wm. H. Trueman.*—Many years ago Dr. J. D. White introduced the practice of making applications of dry arsenic to cavities, letting it remain a short time, blowing it away, and then excavating. For my own part I am extremely afraid of arsenic. I recall now one case in which I used arsenic as an obtundent, and although the pulp remained alive for some years, I had occasion to open the tooth after several years had elapsed and found the pulp dead. It takes a very minute portion of arsenic to kill a pulp. Dr. Flagg says he took one-twelfth grain of arsenic and used it as an application in devitalizing several pulps, and at the end recovered his one-twelfth grain, none of it having been absorbed by the several pulps.

*Dr. Register.*—In years back, I experimented with arsenic as an obtunder and found it failed me. Some twenty years ago I used aromatic sulphuric acid as an obtundent and found it effective. At that time, Dr. Litch suggested to me to use the pure acid: it could do no damage. I tried it, and use it to this day.

*Dr. Burchard.*—Have you noted sinking fillings, softened dentine perforated roots following its use?

*Dr. Register.*—No; no damage whatever has been noted.

*Dr. Darby.*—There is an admirable and most effective agent or preparation which has not been mentioned this evening. It is a mixture of potassium hydrate and carbolic acid in equal parts; commercially the preparation is known as Robinson's remedy. A short period of pain follows its application, much less, however, than when zinc chloride is used. In a few minutes the dentine is insensitive and excavation may be accomplished. Zinc chloride, a very positive obtunder, may produce extreme suffering. I have used sulphuric acid full strength as an obtunder when the fifty-per-cent. solution has failed to obtund.

I find it particularly useful in those labial cavities which are so often exquisitely sensitive. The application sometimes causes pain, but it certainly obtunds, and very promptly too.

*Dr. Huey.*—Perhaps the most annoying and obstinate cases of dentinal hypersensitivity which call for relief are those areas upon the necks of teeth left bare by the recession of the gum, without any evidences of caries being present. The cementum is abraded until worn off, and the exposed dentine is extremely sensitive. Many years ago Dr. Guilford suggested to me the use of a mixture of equal parts of sodium hydrate and carbolic acid. This is very similar to Robinson's remedy, which contains the potassium hydrate. Mixed in equal parts, a gelatinous mass forms, which may be diluted with alcohol. The combination of the sodium and carbolic acid is active, attended by the evolution of much heat. I find this agent for the class of cases named invariably successful.

The painful stage is very short and the anlagesia is complete. To prevent the contact of the obtundent with the gum, I utilize a little wrinkle shown by Dr. Bratt at a meeting of the Pennsylvania State Dental Society two years ago. He uses this to prevent the access of moisture to cervical cavities while filling them with soft foil. A number of very small pieces of bibulous paper are rolled together until they have the size and form of a grain of oats. One end of the roll is tucked under the gum margin between the teeth, and the rest gradually worked under the margin until the other end is beneath the gum in the opposite proximal space. This effectually prevents the contact of the powerful mixture with the gum, if ordinary care be used. For general obtunding I depend upon thorough desiccation.

*Dr. Inglis.*—For some years I practised in Rio Janeiro, and had exceptional opportunities for noting the ill effects of arsenic indiscriminately applied. The Brazilian dentists appear to apply arsenic in superficial cavities as an obtundent, and, as a consequence, dead pulps are very, very common. I can testify to the efficacy of the potassium hydrate and carbolic acid prescription.

I have used lactic acid in the cases for which sulphuric acid has been recommended. It does not corrode the broaches.

*Dr. James Truman.*—Personally I am opposed to the indiscriminate use of powerful acids and escharotics. It must be remembered that in dealing with dentine we are dealing with a vital tissue. As the essayist pointed out, the basis substance of the dentine is penetrated by protoplasmic filaments which are in direct union with the boundary cells of the pulp, and when these protoplasmic filaments are irritated, how can we limit the extent or depth of the irritation? Such agents as sodium and potassium hydrate are very penetrating in effect. The anatomy and physiology of the tissue operated upon must be considered, and we can then see that we cannot limit and define the extent of ultimate irritation which may be induced. A quarter of a century ago I was convinced that the hypersensitivity found in the dentine of carious teeth was due to an acid condition in the parts, and at that time recommended that applications of sodium bicarbonate be used to neutralize the acid, and thus remove the source of irritation; the results of practice bear out the primary opinion, for the alkaline salt certainly acts as an obtundent, without disturbing the vital portions of the dentine.

*The President.*—Dr. Burchard, will you close the discussion?

*Dr. Burchard.*—I have made no notes for discussion, but there are a few points which call for some comment. As to the omission of eucaine from my paper, it was deliberate, as was also not mentioning tropacocaine, an agent which also produces local hyperæmia, instead of the anæmia as cocaine. The latter was named as the representative of a class. Dr. Porter takes me to task for recognizing the chemical factor as the ultimate one in this connection, and certainly it is, so far as actual inductive science permits us to go. I recognize, of course, the importance of speculative reasoning, but, so far as technical matters are concerned, I prefer to set aside the profound interest which I have always felt in speculative philosophy and the formulating of hypotheses as to scientific matters, and deal directly with those things which are actually demonstrable. I did not touch at all upon speculative matters in the essay, omitting even mention of the theories as to the nature of anæsthesia, and among these latter the partially demonstrable premise of the semi-coagulation theory of anæsthesia, with which no doubt we are all familiar. I did not even classify certain remedies in the group producing the condition named by Liebreich, "anæstheica dolorosa." The essay is purely clinical and not in the least speculative. I think, however, that if Dr. Porter will carry his speculation in a direct line he will come to see that the entire science of pharmacology is inseparable from chemistry, which applies also to even the science of biology. This much for speculative reasoning. In regard to the use of arsenic as an obtunding agent, my opinion, based upon all sound clinical evidences is that it should be absolutely condemned and excluded. Its effects are necrotic and deeply so. It should never be used unless the death of the pulp is deliberately desired. I view even Fowler's solution with suspicion, and certainly should not use it until clinical records are much more extensive.

I am at a loss to understand how the effects named by Dr. Freeman as following applications of sulphuric acid could be brought about unless the tooth were actually drenched with the acid. The effect of sulphuric acid is chemically self-limited, and it would require a great deal of acid or a great deal of carelessness to bring about the conditions he describes. I have used it quite freely in root-canals, and have even carried it through the apex of the root, and have had no trouble; far from having its effect wide-spread, it is difficult and tedious to get the acid to accomplish the limited dentine destruction desired.

I had hoped in the essay to call more definite and pronounced attention to the studies of Dr. Truman as to the relative coagulating power of chemical agents; the subject has an important clinical bearing.

Henry H. Burchard,
*Editor Academy of Stomatology.*

